# Psoriasis and Metabolic Disorders: A Comprehensive Meta-Analysis of Million Adults Worldwide

**DOI:** 10.7759/cureus.52099

**Published:** 2024-01-11

**Authors:** Waleed A Alajroush, Abdulelah I Alrshid, Ahmed H Alajlan, Yazeed B Alsalamah, Mohammed I Alhumaidan, Abeer I Alhoumedan, Mansour I Alrasheed, Yazeed A Alowairdhi, Fatimah Alowirdi, Abdulaziz Z Aljoufi, Duhaim S Alsubaie, Nasser H Alarfaj

**Affiliations:** 1 Department of Pediatric Dermatology, King Abdulaziz Medical City, Riyadh, SAU; 2 College of Medicine, King Saud University, Riyadh, SAU; 3 College of Medicine, Department of Clinical Sciences, Dar Al Uloom University, Riyadh, SAU; 4 Department of Family Medicine, Ministry of National Guard-Health Affairs. King Abdullah International Medical Research Center, Riyadh, SAU; 5 Department of Medicine, King Saud Bin Abdulaziz University for Health Sciences College of Medicine, Riyadh, SAU; 6 Department of Family Medicine, King Faisal Specialist Hospital & Research Centre, Riyadh, SAU; 7 Department of Dermatology, King Abdulaziz Medical City, Riyadh, SAU

**Keywords:** hyperlipidemia, hypertension, diabetes, obesity, metabolic syndrome, meta-analysis, psoriasis, metabolic disorders

## Abstract

Psoriasis, a chronic inflammatory skin condition, and metabolic disorders, such as obesity, diabetes, and dyslipidemia, have long been recognized as distinct clinical entities. However, emerging evidence suggests a complex bidirectional relationship between these seemingly unrelated conditions. Psoriasis is characterized by an accelerated skin cell turnover, resulting in the formation of erythematous plaques with silvery scales. Metabolic disorders, on the other hand, encompass a range of conditions associated with abnormal metabolic processes, including insulin resistance, dyslipidemia, and chronic low-grade inflammation. It is intriguing to note that psoriasis is commonly associated with several metabolic comorbidities, with a higher prevalence observed in individuals with obesity, type 2 diabetes, and metabolic syndrome. Mounting evidence suggests that chronic inflammation plays a pivotal role in both psoriasis and metabolic disorders. Shared inflammatory mediators, such as tumor necrosis factor-alpha (TNF-α), interleukin-6 (IL-6), and C-reactive protein (CRP), have been implicated in the pathogenesis of both conditions. Moreover, adipose tissue-derived hormones, known as adipokines, including leptin and adiponectin, exert modulatory effects on immune responses and may contribute to the link between psoriasis and metabolic abnormalities. Following Preferred Reporting Items for Systematic Reviews and Meta-Analyses (PRISMA) guidelines, a comprehensive search across databases identified 16 eligible studies (1975-2023), totaling 6,623,379 participants. Inclusion criteria encompassed peer-reviewed observational studies focusing on adults and specified outcomes. Data extraction, quality assessment (Newcastle-Ottawa scale (NOS)), meta-analyses, and heterogeneity evaluations were conducted using rigorous methods. Psoriasis displayed a significant association with diabetes mellitus (DM, 18% increased incidence), hypertension (HTN, 35%), hyperlipidemia (19%), and obesity (25%). Substantial heterogeneity was observed in meta-analyses, particularly for DM. The NOS indicated varied study quality, with some studies categorized as a high or moderate risk of bias.

## Introduction and background

Psoriasis is a chronic inflammatory skin disease that affects about 2-3% of the global population [1]. It is characterized by red, scaly, and itchy plaques that can occur anywhere on the body, but most commonly on the elbows, knees, scalp, and lower back [1-2]. Psoriasis can have a significant impact on the quality of life of patients, affecting their physical, psychological, and social well-being [3]. Psoriasis is also associated with various comorbidities, especially metabolic disorders, such as diabetes mellitus (DM), hypertension (HTN), hyperlipidemia, and obesity [4-5]. These conditions share common pathophysiological mechanisms with psoriasis, such as chronic inflammation, oxidative stress, insulin resistance, and dyslipidemia. Moreover, metabolic disorders can increase the risk of cardiovascular diseases, which are the leading cause of mortality in patients with psoriasis [6-8]. The prevalence and incidence of metabolic disorders in patients with psoriasis have been investigated in several observational studies, but the results are inconsistent and heterogeneous.

Some studies have reported a higher risk of metabolic disorders in patients with psoriasis compared to the general population, while others have found no significant difference or even a lower risk [7-10]. The reasons for these inconsistencies may include differences in study design, population characteristics, diagnostic criteria, confounding factors, and outcome measures. Therefore, there is a need for a systematic review and meta-analysis to synthesize the available evidence and provide a reliable estimate of the association between psoriasis and metabolic disorders. A systematic review and meta-analysis are a type of review that uses repeatable methods to find, select, and synthesize all relevant studies on a specific topic. It can reduce bias, increase precision, and resolve the uncertainty by combining the results of individual studies.

The aim of this systematic review and meta-analysis is to answer the following research question: What is the association between psoriasis and the outcomes of interest, namely, DM, HTN, hyperlipidemia, and obesity, in adult patients?

## Review

Materials and methods

We conducted this systematic review and meta-analysis according to the Preferred Reported Items of Systematic Review and Meta-analysis (PRISMA) guidelines 2020 [11]. 

Search Strategy

A comprehensive search of electronic databases including PubMed, EMBASE, and Cochrane Library was conducted from inception to December 2023, with no language restrictions. The search strategy employed a combination of Medical Subject Headings (MeSH) terms and keywords related to psoriasis, DM, HTN, hyperlipidemia, and obesity. A manual search of reference lists from relevant articles and reviews was also performed to identify additional studies.

The* *inclusion and exclusion criteria of the study are presented in Table 1.* *

**Table 1:** Study selection criteria. In our review, we included studies published in English from 1975 to 2023, which focused on patients with psoriasis and metabolic disorders that met the inclusion criteria.

Inclusion criteria	Exclusion criteria
Original research articles published in peer-reviewed journals	Studies without a clear comparison group (non-psoriatic individuals)
Studies that investigated the association between psoriasis and the outcomes of interest: diabetes mellitus, hypertension, hyperlipidemia, and obesity are adult patients	Animal studies, case reports, reviews, and conference abstracts
Observational studies (cohort, case-control, case series, or cross-sectional designs) reporting relevant data	Studies with insufficient data for extraction
Studies with human participants	Children or adolescent patients

Data Extraction

Two independent reviewers conducted the initial screening of titles and abstracts based on the inclusion and exclusion criteria. Full-text articles of potentially relevant studies were then assessed for eligibility. Disagreements were resolved through discussion or consultation with a third reviewer. Data extraction included study characteristics (study design, study site and time, total sample size criteria, duration of follow-up, age, and gender), and relevant outcome data (risk ratios (RRs) or odds ratios (ORs) and 95% confidence intervals (CIs)).

Quality Assessment

We used the Newcastle-Ottawa scale (NOS) [12] to assess the methodological quality of cohort, case-control, and cross-sectional studies. The NOS awards stars to each study based on selection, comparability, exposure, and outcome criteria. The maximum stars are nine for cohort and case-control studies and 10 for cross-sectional studies. Studies with more than six or seven stars are high quality, while studies with less than four or five stars are low quality.

Statistical Analysis

Meta-analyses were performed using Stata Statistical Software release 17 (StataCorp., 2021, College Station, TX: StataCorp LLC), and random-effect model and pooled RRs or ORs with 95% CIs were calculated for each outcome. Heterogeneity was assessed using the Q-test and the tau-squared statistic, with values greater than 50% indicating substantial heterogeneity.

Publication Bias

Publication bias was examined through the inspection of funnel plots. The asymmetry in funnel plots was considered as a potential indicator of publication bias.

Ethics and Registration

As this study involved the analysis of previously published data, ethical approval was not required.

Results

Search Results

**Figure 1:**
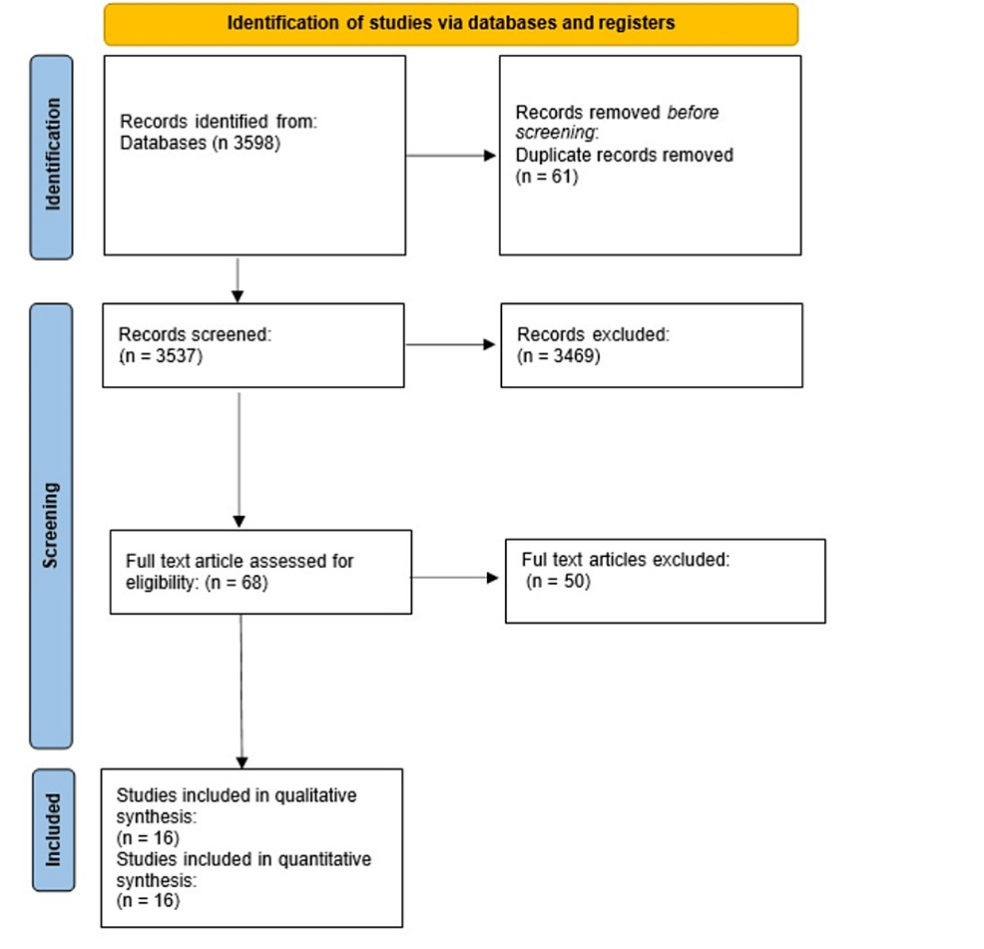
PRISMA flow diagram of the inclusion/exclusion criteria PRISMA: Preferred Reporting Items for Systematic Reviews and Meta-Analyses; n: number

The initial electronic database search yielded a total of 3,598 articles. After removing duplicates and conducting title and abstract screening, 68 studies were eligible for full-text assessment. Following the application of inclusion and exclusion criteria, 16 studies were included in the systematic review and meta-analysis. More details regarding the searching process in the PRISMA flow diagram are presented in Figure 1.

Characteristics of the Included Studies

The included studies had a total of 6,623,379 participants and were published between 1975 and 2023. Study designs comprised 12 cohort studies, one case-control study, and three cross-sectional studies. Participants in these studies varied in age, gender, and habit states. The summary of the included studies and patient characteristics is detailed in Table 2.

**Table 2:** Summary of the included studies and patient characteristics NR: not recorded

Study ID	Study design	Study site and time	Total sample size	Duration of follow-up	Age		Sex	
					Mean	SD	Male (n)%	Female (n)%
Armesto et al., 2012 [13]	Cohort	Asturias and Cantabria (northern Spain)	1,322	NR	47.43	15.71	350 (53)	311 (47)
Binazzi et al., 1975 [14]	Cohort	Umbria, Italy. From December 1972 and December 1974	200	NR	NR	NR	127 (63.5)	73 (36.5)
Lynch et al., 1976 [15]	Cohort	Michigan. From 1963 to 1965	346	NR	47	NR	NR	NR
Azfar et al., 2012 [16]	Cohort	We used the Health Improvement Network (THIN) data collected from 2003 through October 2008	108,132	1 year	46.03	16.62	52 420 (48.4	55 679 (51.51)
Brauchli et al., 2008 [17]	Cohort	UK. From January 1, 1994 and 31 December 2005	36,702	Followed up until they developed a first-time diagnosis of DM, died, or follow-up in the medical record ended, whichever came first.	NR	NR	NR	NR
Chiu et al., 2020 [18]	Cohort	Taiwan. From 2000 to December 31, 2013	9,735	The follow‑up period was from 2000 to the date of the outcome of interest, date of death, or December 31, 2013.	55.4	14.8	16,527 (52.1)	15,170 (47.9)
Dubreuil et al., 2013 [19]	Cohort	UK (19862010)	74,635	Participants were followed until they developed diabetes, died or the follow-up ended, whichever came first.	49.2	17.1	NR	30,330 (51.2)
Khalid et al., 2013 [20]	Cohort	Denmark. From January 1, 1997 to December 31, 2009	4,614,807	Maximum follow-up of 13 years. From January 1, 1997 to December 31, 2009.	45.3	16.9	22,631 (49.4)	22,631 (49.4)
lee et al., 2014 [21]	Cohort	Taiwan. January 1, 1999, and December 31, 2008	14,158	Patients were followed up from the index date until the earliest date of the development of an outcome of interest, the date of death, or December 31, 2008.	42.39	19.45	3,578 (54.12)	NR
Eder et al.,2017 [22]	Cohort	NR	1,305	The mean (SD) duration of follow up was 9.1 (9.1) years.	53.7	13.9	NR	470 (44.1)
Wan et al., 2017 [23]	Cohort	UK	NR	4 years	45	13.3	4,088 (50.32)	4,036 (49.68)
Soloman et al., 2010 [24]	Cohort	British Columbia (BC), Canada. From January 1, 1996 through 31 December 2006	40,346	Subjects were followed up until they experienced an outcome, died, left BC or follow- up ended (December 31, 2006).	50	17	NR	20,038 (50)
Shapiro et al., 2007 [25]	Case-control	Israel. Between 1997 and 2004	1,625,132	NR	41.28	18.8	23,064(50)	23,031 (50)
Qureshi et al., 2009 [26]	Cross-sectional	USA	78,061	The follow-up rate exceeded 90% for each two-year period.	36.4	NR	NR	NR
Cohen et al., 2008 [27]	Cross-sectional	Israel	16,851	NR	44.7	16.1	3,196 (47.1)	3,588 (52.9)
Dreiher et al., 2013 [28]	Cross-sectional	Israel	1,647	NR	53.8	14.1	249 (45.4%)	NR

Primary Outcome

**Figure 2:**
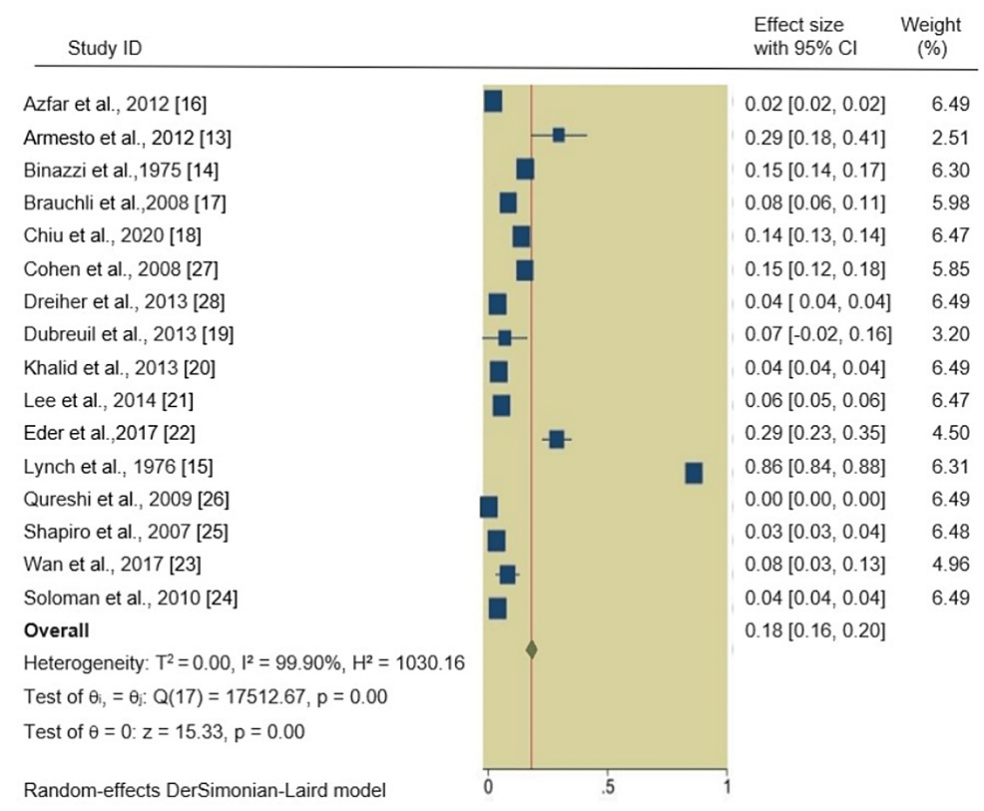
Summary of the included studies and patient characteristics

DM in psoriatic patients: We found a significant association between psoriasis and DM2. The pooled RR of DM2 in psoriatic patients was 1.18 (95% CI 1.16, 1.20), indicating an 18% increased incidence of DM2 in psoriatic patients compared to non-psoriatic patients. The forest plot of the primary outcome is shown in Figure 2

Secondary Outcomes

**Figure 3:**
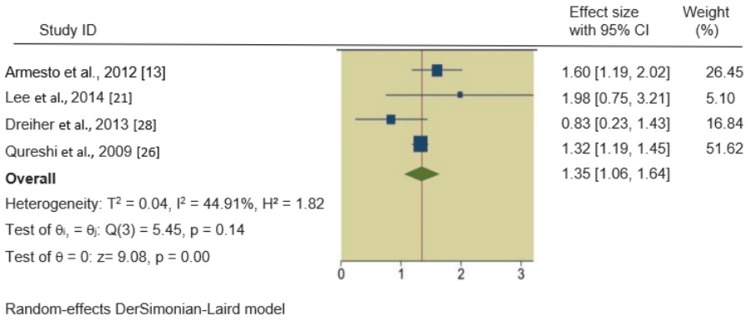
Forest plot of the incidence of hyperlipidemia in psoriatic patients.

**Figure 4:**
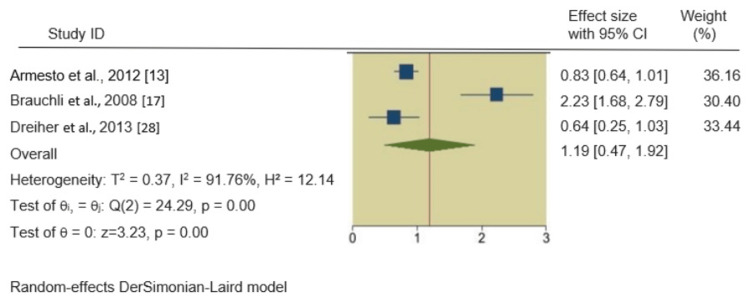
Forest plot of the incidence of obesity in psoriatic patients.

**Figure 5:**
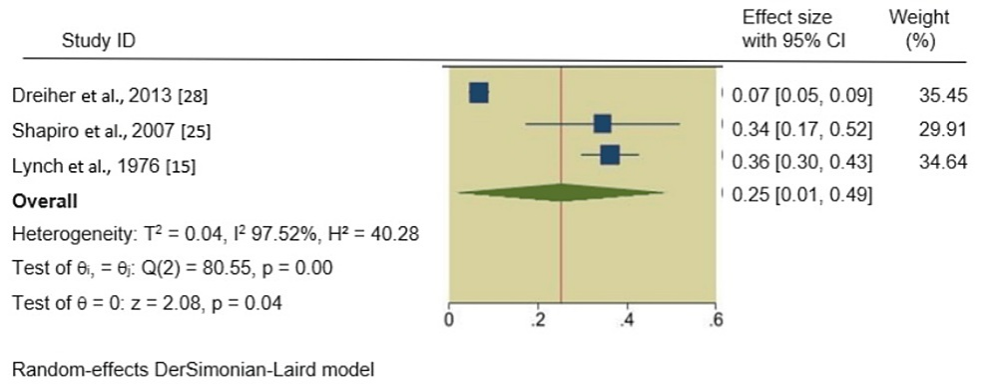
Forest plot of the incidence of obesity in psoriatic patients.

HTN, hyperlipidemia, and obesity in psoriatic patients: We also found significant associations between psoriasis and HTN, hyperlipidemia, and obesity. The pooled RRs of these outcomes in psoriatic patients were 1.35 (95% CI 1.06, 1.64), 1.19 (95% CI 0.47, 1.92), and 1.25 (95% CI 1.01, 1.49), respectively, indicating a 35%, 19%, and 25% increased incidence of these outcomes in psoriatic patients compared to non-psoriatic patients. The forest plots of the secondary outcomes are shown in Figures 3-5.

Heterogeneity Assessment

In all four meta-analyses, heterogeneity was rigorously examined. For the primary outcome, DM in psoriatic patients, the heterogeneity was exceptionally high (I^2^ = 99.90%). No significant heterogeneity was observed in the HTN analysis (I^2^ = 44.91%), while severe heterogeneity was evident in the hyperlipidemia (I^2^ = 91.76%) and obesity (I^2^ = 97.52%) analyses. Q-square p-values were consistently significant (p = 0.00, p = 0.14) across all outcomes, confirming the presence of substantial heterogeneity.

Quality Assessment

The NOS [12] was employed to evaluate the quality of the included studies across cohorts, case control studies, and cross-sectional designs.

Cohort studies: Three studies (Armesto et al. (2012) [13], Binazzi et al. (1975) [14], and Lynch et al. (1976) [15]) were identified as high risk due to non-compliance with most NOS criteria, particularly in selection, comparability, and outcome reporting. Nine studies (Azfar et al. (2012) [16], Brauchli et al. (2008) [17], Chiu et al. (2020) [18], Dubreuil (2013) [19], Khalid et al. (2013) [20], Lee et al. (2014) [21], Eder et al. (2017) [22], Wan et al. (2017) [23], and Soloman et al. (2010) [24]) demonstrated low risk of bias, meeting the majority of the NOS criteria.

Case control study: Shapiro et al. (2007) [25] was deemed to have a low risk of bias, meeting most of the NOS criteria.

Cross-sectional studies: Three studies (Qureshi et al. (2009) [26]) was categorized as high risk due to non-compliance with the key NOS criteria. Cohen et al. (2008 [27]) demonstrated low risk of bias, meeting all or most of the NOS criteria. Dreiher et al. (2013 [28]) was considered a moderate-risk study for meeting some selection and comparability criteria but lacking in outcome reporting. Table 3 illustrates the detailed data of the NOS assessment.

**Table 3:** NOS results of the included studies. NOS: Newcastle-Ottawa scale

Cohort studies								
Study ID	Selection				Comparability	Outcome		
	Representativeness of the exposed cohort	Selection of the non-exposed cohort	Ascertainment of exposure	Demonstration that the outcome of interest was not present at the start of the study	Comparability of cohorts on the basis of the design or analysis	Assessment of outcome	Was follow-up long enough for outcomes to occur?	Adequacy of follow-up of cohorts
Armesto et al., 2012 [13]	+	+	-	-	-	+	-	+
Binazzi et al., 1975 [14]	+	-	+	+	-	+	-	-
Lynch et al., 1976 [15]	+	+	-	-	+	-	-	-
Azfar et al., 2012 [16]	+	+	+	+	++	+	-	+
Brauchli et al., 2008 [17]	+	+	+	+	++	+	+	-
Chiu et al.,2020 [18]	+	+	+	+	+	+	+	+
Dubreuil 2013 [19]	+	+	+	+	++	+	+	+
Khalid et al., 2013 [20]	+	+	+	+	++	+	+	+
Lee et al.,2014 [21]	+	+	+	+	+	+	+	+
Eder et al., 2017 [22]	+	-	+	+	-	+	+	+
Wan et al., 2017 [23]	+	+	+	+	++	+	+	-
Soloman et al., 2010 [24]	+	+	+	+	++	+	+	+
Case control studies								
Study ID	Selection				Comparability	Exposure		
	Is the case definition adequate	Representativeness of the cases	Selection of controls	Definition of controls	Comparability of cases and controls on the basis of the design or analysis	Ascertainment of exposure	Same method of ascertainment for cases and controls	Non-response rate
Shapiro et al., 2007 [25]	-	+	+	+	-	+	+	+
Cross-sectional studies								
Study ID	Selection				Comparability		Outcome	
	Representativeness of the sample	Non-respondents	Ascertainment of the exposure		The subjects in different outcome groups are comparable, based on the study design or analysis. Confounding factors are controlled.		Assessment of the outcome	Statistical test
Qureshi et al., 2009 [26]	-	+	-		-	-	-	+
Cohen et al., 2008 [27]	+	+	+		+	+	+	+
Dreiher et al., 2013 [28]	+	+	+		-	-	+	+

Discussion

Interpretation of Findings

The systematic review and meta-analysis aimed to assess the association between psoriasis and metabolic disorders, specifically DM, HTN, hyperlipidemia, and obesity. Our study revealed a significant 18% increase in the incidence of DM in psoriatic patients. In addition, psoriatic patients exhibited a significant risk of HTN, hyperlipidemia, and obesity by 35%, 19%, and 25%, respectively. These findings confirm the elevated risk of developing metabolic diseases in psoriasis. This association may be attributed to various mechanisms in psoriasis, such as the PSORS1 locus, which encodes the major histocompatibility complex class I molecule HLA-Cw6. This locus is the strongest genetic risk factor for psoriasis and is also linked to type 2 diabetes and celiac disease [29-31]. Psoriasis is characterized as a type 17 immune-mediated disease, wherein the activation of the innate and adaptive immune system leads to the production of pro-inflammatory cytokines, including tumor necrosis factor-alpha (TNF-α), interleukin-6 (IL-6), interleukin-17 (IL-17), and interleukin-23 (IL-23) [32-34]. These cytokines can induce systemic inflammation and oxidative stress, impairing the function of the endothelium, pancreas, and liver, ultimately leading to insulin resistance and dyslipidemia. Insulin resistance and dyslipidemia, in turn, can further exacerbate inflammation and oxidative stress, creating a vicious cycle that increases the risk of DM, HTN, hyperlipidemia, and obesity.

Comparison with Previous Studies

Our findings align with previous systematic reviews and meta-analyses that have reported similar associations between psoriasis and metabolic disorders. A recent meta-analysis by Yuan et al. (2022) [9] included five studies and found that psoriasis was associated with an increased risk of DM (RR = 1.54, 95% CI: 1.43-1.67). Another meta-analysis by Liu et al. (2022) [7] indicated that psoriasis was associated with a 32% increased risk of metabolic syndrome, with a 95% CI of 0.26-0.38. The pooled estimates from our meta-analysis not only confirm the results of the previous studies but also provide additional data on outcomes other than DM.

Clinical or Practical Implications

The implications of our findings are important for the clinical management, prevention, and prognosis of psoriasis and metabolic disorders. Patients with psoriasis should be regularly screened and monitored for metabolic disorders and receive appropriate treatment and lifestyle interventions to reduce their risk of cardiovascular diseases. Clinicians should also consider the potential impact of psoriasis treatment on metabolic disorders, as some therapies may have beneficial or adverse effects on glucose and lipid metabolism. For example, systemic therapies, such as methotrexate, cyclosporine, and biologics, may improve the metabolic profile of patients with psoriasis by reducing the inflammation and oxidative stress, while topical therapies, such as corticosteroids, may worsen the metabolic profile by inducing skin atrophy and systemic absorption [35-36]. Furthermore, patients with metabolic disorders should be aware of the possible increased risk of developing psoriasis and seek early diagnosis and treatment if they experience any skin symptoms.

Strengths and Limitations

Our review has several strengths and limitations that should be acknowledged. The strengths include the comprehensive and systematic search of multiple databases, the use of rigorous inclusion and exclusion criteria, the quality assessment of the included studies using the NOS, the meta-analysis using a random-effect model, and the assessment of heterogeneity and publication bias. The limitations include the heterogeneity and variety of the included studies in terms of study design, population characteristics, diagnostic criteria, confounding factors, and outcome measures. The heterogeneity was substantial for all outcomes, as indicated by the high I^2^ values. These analyses were limited by the availability and quality of the data and could not account for all the potential sources of heterogeneity. Therefore, the results should be interpreted with caution, and generalizability may be limited. Another limitation is the possibility of publication bias, as suggested by the asymmetrical funnel plots. This may indicate that some studies with negative or null results were not published or not identified by our search, which could lead to an overestimation of the association. However, publication bias is difficult to detect and may be influenced by other factors, such as sample size and study quality.

Recommendations for Future Research

Based on our findings and limitations, we recommend the following directions for future research: More high-quality observational studies, especially prospective cohort studies, are needed to confirm and clarify the association between psoriasis and metabolic disorders and to control for potential confounding factors, such as age, sex, smoking, alcohol, and other comorbidities. More standardized and validated diagnostic criteria and outcome measures are needed to improve the comparability and consistency of the studies and to reduce the heterogeneity and variability of the results. More studies are needed to investigate the causal mechanisms and pathways underlying the association between psoriasis and metabolic disorders and to identify the potential biomarkers and mediators of this association. More studies are needed to evaluate the effect of psoriasis treatment on metabolic disorders and to compare the efficacy and safety of different therapies on glucose and lipid metabolism. More studies are needed to assess the impact of metabolic disorders on psoriasis and to explore the bidirectional relationship between these conditions.

## Conclusions

This systematic review and meta-analysis provide compelling evidence supporting a significant association between psoriasis and metabolic disorders, including diabetes mellitus, hypertension, hyperlipidemia, and obesity in adult patients. The findings underscore the importance of regular screening and monitoring for metabolic disorders in individuals with psoriasis and highlight the potential bidirectional impact of these conditions. Clinicians should be vigilant in managing both psoriasis and metabolic disorders concurrently to improve patient outcomes. However, caution is warranted in interpreting the results due to substantial heterogeneity among included studies and potential publication bias. Future research should focus on high-quality prospective cohort studies, standardized diagnostic criteria, and elucidating the underlying mechanisms of this association, as well as exploring the impact of psoriasis treatment on metabolic outcomes.

## References

[1] Dowlatshahi EA, van der Voort EA, Arends LR, Nijsten T (2013). Markers of systemic inflammation in psoriasis: a systematic review and meta-analysis. Br J Dermatol.

[2] Boehncke W, Schön M (2015). Psoriasis. Lancet.

[3] Trettin B, Feldman SR, Andersen F, Danbjørg DB, Agerskov H (2020). A changed life: the life experiences of patients with psoriasis receiving biological treatment. Br J Dermatol.

[4] Bissonnette R, Searles G, Landells I (2009). The AWARE study: methodology and baseline characteristics. J Cutan Med Surg.

[5] Han EJ, Yoo SA, Kim GM, Hwang D, Cho CS, You S, Kim WU (2016). GREM1 Is a key regulator of synoviocyte hyperplasia and invasiveness. J Rheumatol.

[6] Zhou Q, Mrowietz U, Rostami-Yazdi M (2009). Oxidative stress in the pathogenesis of psoriasis. Free Radic Biol Med.

[7] Ju Y, Pei H, Kang N (2022). Prevalence and potential risk factors of chronic pruritus among community middle-aged and older population in Beijing, China. J Eur Acad Dermatol Venereol.

[8] Robertson RP (2004). Chronic oxidative stress as a central mechanism for glucose toxicity in pancreatic islet beta cells in diabetes. J Biol Chem.

[9] Garcia Salinas R, Jaldin Cespedes R, Gomez RA, Aguerre D, Sommerfleck F (2022). Non-radiographic axial spondyloarthritis in South America. Burden of disease and differential features with respect to ankylosing spondylitis at time of diagnosis. A comprehensive analysis with a focus on images. Int J Rheum Dis.

[10] Fenton A, Elliott E, Shahbandi A (2020). Medical students' ability to diagnose common dermatologic conditions in skin of color. J Am Acad Dermatol.

[11] Page MJ, McKenzie JE, Bossuyt PM (2021). The PRISMA 2020 statement: an updated guideline for reporting systematic reviews. BMJ.

[12] Higgins JP, Altman DG, Gøtzsche PC (2011). The Cochrane Collaboration's tool for assessing risk of bias in randomised trials. BMJ.

[13] Boyce AE, McGrath JA, Techanukul T, Murrell DF, Chow CW, McGregor L, Warren LJ (2012). Ectodermal dysplasia-skin fragility syndrome due to a new homozygous internal deletion mutation in the PKP1 gene. Australas J Dermatol.

[14] Binazzi M, Calandra P, Lisi P (1975). Statistical association between psoriasis and diabetes: further results. Arch Dermatol Res (1975).

[15] Lynch P (1967). Psoriasis and blood sugar levels. Arch Dermatol.

[16] Azfar RS, Seminara NM, Shin DB, Troxel AB, Margolis DJ, Gelfand JM (2012). Increased risk of diabetes mellitus and likelihood of receiving diabetes mellitus treatment in patients with psoriasis. Arch Dermatol.

[17] Brauchli YB, Jick SS, Meier CR (2008). Psoriasis and the risk of incident diabetes mellitus: a population-based study. Br J Dermatol.

[18] Chiu HY, Hung CJ, Muo CH, Fan KC, Sung FC (2020). The bidirectional association between type 2 diabetes and psoriasis: Two retrospective cohort studies. Indian J Dermatol Venereol Leprol.

[19] Malbos S, Urena-Torres P, Cohen-Solal M, Trout H, Lioté F, Bardin T, Ea HK (2014). Sodium thiosulphate treatment of uraemic tumoral calcinosis. Rheumatology (Oxford).

[20] Rosenstock J, Balas B, Charbonnel B, Bolli GB, Boldrin M, Ratner R, Balena R (2013). The fate of taspoglutide, a weekly GLP-1 receptor agonist, versus twice-daily exenatide for type 2 diabetes: the T-emerge 2 trial. Diabetes Care.

[21] Sako EY, Famenini S, Wu JJ (2014). Bariatric surgery and psoriasis. J Am Acad Dermatol.

[22] Eder L, Chandran V, Cook R, Gladman DD (2017). The risk of developing diabetes mellitus in patients with psoriatic arthritis: a cohort study. J Rheumatol.

[23] Kim JY, Kozlow JH, Mittal B, Moyer J, Olenecki T, Rodgers P (2018). Guidelines of care for the management of cutaneous squamous cell carcinoma. J Am Acad Dermatol.

[24] Solomon DH, Love TJ, Canning C, Schneeweiss S (2010). Risk of diabetes among patients with rheumatoid arthritis, psoriatic arthritis and psoriasis. Ann Rheum Dis.

[25] Bercovitch L, Long TP (2007). Dermatoethics: a curriculum in bioethics and professionalism for dermatology residents at Brown Medical School. J Am Acad Dermatol.

[26] Qureshi AA, Choi HK, Setty AR, Curhan GC (2009). Psoriasis and the risk of diabetes and hypertension: a prospective study of US female nurses. Arch Dermatol.

[27] Cohen AD, Dreiher J, Shapiro Y, Vidavsky L, Vardy DA, Davidovici B, Meyerovitch J (2008). Psoriasis and diabetes: a population-based cross-sectional study. J Eur Acad Dermatol Venereol.

[28] Dreiher J, Freud T, Cohen AD (2013). Psoriatic arthritis and diabetes: a population-based cross-sectional study. Dermatol Res Pract.

[29] Shevchenko A, Valdes-Rodriguez R, Yosipovitch G (2018). Causes, pathophysiology, and treatment of pruritus in the mature patient. Clin Dermatol.

[30] Sarandi E, Krueger-Krasagakis S, Tsoukalas D (2023). Psoriasis immunometabolism: progress on metabolic biomarkers and targeted therapy. Front Mol Biosci.

[31] Pietrzak A, Bartosińska J, Chodorowska G, Szepietowski JC, Paluszkiewicz P, Schwartz RA (2021). Metabolic syndrome in psoriasis: a review. Acta Dermatovenerol Croat.

[32] Brembilla NC, Boehncke WH (2023). Revisiting the interleukin 17 family of cytokines in psoriasis: pathogenesis and potential targets for innovative therapies. Front Immunol.

[33] Łuczaj W, Moneta-Wielgos J, Stawczyk-Macieja M (2022). Association between psoriasis and chronic kidney disease: a population-based cross-sectional study. Acta Derm Venereol.

[34] Husted JA, Thavaneswaran A, Chandran V, Eder L, Rosen CF, Cook RJ, Gladman DD (2011). Cardiovascular and other comorbidities in patients with psoriatic arthritis: a comparison with patients with psoriasis. Arthritis Care Res (Hoboken).

[35] Nijsten T, Wakkee M (2009). Complexity of the association between psoriasis and comorbidities. J Invest Dermatol.

[36] Cohen AD, Sherf M, Vidavsky L, Vardy DA, Shapiro J, Meyerovitch J (2008). Association between psoriasis and the metabolic syndrome. A cross-sectional study. Dermatology.

